# Efficacy and Safety of Hyaluronic Acid Fillers for Midface Augmentation: A Systematic Review and Meta-Analysis

**DOI:** 10.3390/medicina61101823

**Published:** 2025-10-11

**Authors:** Alaa Safia, Uday Abd Elhadi, Shlomo Merchavy, Ramzy Batheesh, Naji Bathish

**Affiliations:** 1 Department of Otolaryngology, Ziv Medical Center, Safed 1311001, Israel; udayabdi510@gmail.com (U.A.E.); shlomo.m@ziv.health.gov.il (S.M.); 2 Department of Dermatology, Rambam Health Care Campus, Haifa 3109601, Israel; ramzy_ra512@hotmail.com; 3 Department of Dermatology, Ziv Medical Center, Safed 1311001, Israel; najibathish@gmail.com

**Keywords:** hyaluronic acid, dermal fillers, midface augmentation, meta-analysis, aesthetic outcomes

## Abstract

*Background and Objectives*: Hyaluronic acid (HA) fillers are commonly used for midface augmentation because of their biocompatibility and reversibility. Nonetheless, discussions continue about their effectiveness and safety relative to other options. This systematic review and meta-analysis assess the effectiveness, duration, and side effects of HA fillers in midface volume restoration. *Materials and Methods*: Following PRISMA guidelines, a thorough search was performed on PubMed, CENTRAL, Web of Science, Scopus, and EMBASE up to March 2025. The review included randomized controlled trials (RCTs) that compared HA fillers with controls, such as placebo or alternative treatments, for midface augmentation. *Results*: A total of fourteen studies were included in the review, and five studies in the statistical analysis. Analysis of five RCTs involving 748 participants showed a higher and significant difference in GAIS responder rates between HA and control groups (RR = 3.27, 95% CI: 2.26–4.75, *p* = 0.79; I^2^ = 95%). GAIS scores at 4, 8, and 24 weeks demonstrated no notable improvements (all *p* > 0.05). Adverse events were rarely reported, and there was no significant rise in moderate-to-severe adverse events associated with HA fillers (RR = 1.70, 95% CI: 0.08–34.55, *p* = 0.73). *Conclusions*: HA fillers used for midface augmentation are generally safe, they have very high midface augmentation and patient satisfaction value, but they might not provide a notable subjective aesthetic benefit over the other fillers. Clinicians need to take into account patient expectations and refine their techniques, all while recognizing the limitations of existing evidence. Future research should include objective volumetric measurements and extend follow-up durations.

## 1. Introduction

Midface aging is characterized by volume loss, soft tissue descent, and skeletal changes, leading to flattening of the malar region, deepening of nasolabial folds, and an overall tired appearance. Injectable fillers, particularly hyaluronic acid (HA)-based products, have become one of the most sought-after minimally invasive treatments for facial rejuvenation [1]. According to the American Society of Plastic Surgeons (ASPS), over 2.7 million soft tissue filler procedures were performed in the United States in 2019, with HA fillers accounting for 2.1 million of these treatments a testament to their widespread acceptance and popularity [2]. The appeal of HA fillers lies in their biocompatibility, reversible nature, and ability to provide immediate, natural-looking volume restoration with minimal downtime [3].

The midface plays a pivotal role in facial aesthetics, and its aging process involves complex interactions between bone resorption, fat redistribution, and skin elasticity loss [1,4]. Several classification systems and aesthetic ideals have been proposed to guide treatment, including the zygomaxillary point [5], the WIZDOM (width of the zygomatic distance of the midface) [6], and the golden ratio (Phi). These frameworks underscore the importance of tailored approaches to midface augmentation, considering variations in patient anatomy, age-related changes, and aesthetic goals [7].

HA, a naturally occurring glycosaminoglycan, is an ideal dermal filler due to its high hydrophilicity, biodegradability, and low immunogenicity [8]. However, commercially available HA fillers differ in concentration, cross-linking density, and viscosity, influencing their longevity, rheological properties, and clinical applications. Various injection techniques—such as submuscular placement in the zygomatic region and subcutaneous deposition in the submalar area—have been explored to optimize outcomes while minimizing complications [9,10]. Despite their favorable safety profile, HA fillers can still lead to adverse events, ranging from transient swelling and bruising to rare but serious complications like vascular occlusion and skin necrosis [11].

Given the increasing demand for midface augmentation and the diversity of HA products and techniques, a systematic evaluation of their efficacy, safety, and longevity is essential. This systematic review aims to synthesize existing evidence on HA fillers for midface volume restoration, assessing patient satisfaction, duration of effect, and complication rates across different formulations and injection methods. By consolidating current knowledge, this review seeks to guide clinicians in making evidence-based decisions to achieve optimal, personalized outcomes for their patients.

## 2. Materials and Methods

### 2.1. Protocol Registration

This systematic review adhered to the Preferred Reporting Items for Systematic Reviews and Meta-Analyses (PRISMA) guidelines [12] and the Cochrane Handbook of Systematic Reviews [13]. The study protocol was registered with the International Prospective Register of Systematic Reviews (PROSPERO) under ID (CRD420251132690).

### 2.2. Data Sources and Search Strategy

A comprehensive search was conducted until March 2024 across five databases (PubMed, CENTRAL, Web of Science, Scopus, EMBASE) without language or date restrictions. The search strategy combined Medical Subject Headings (MeSH) terms and keywords: (“hyaluronic acid”[MeSH] OR “HA filler”[tiab] OR “Juvederm”[tiab]) AND (“midface”[tiab] OR “malar”[tiab]) AND (“augmentation”[tiab] OR “volumization”[tiab]). We employed a comprehensive search strategy to ensure that a broad range of relevant clinical trials was captured in our database searches.

### 2.3. Eligibility Criteria

Studies were included based on the PICO framework:

P: Patients undergoing midface augmentation with HA fillers.

I: HA filler injection (any formulation, e.g., Juvederm Voluma, Restylane Lyft).

C: Comparative studies (e.g., different other fillers or nonstandard HA products, injection techniques, or placebo).

O: Primary outcomes (patient satisfaction, GAIS responders, adverse events) and secondary outcomes (aesthetic improvement scales).

Studies involving animal research, reviews, case reports, and conference abstracts were excluded from the analysis. Additionally, studies that combined HA fillers with other therapies, such as botulinum toxin, were also excluded to ensure a focused evaluation of HA filler outcomes. These exclusion criteria were implemented to maintain the relevance and quality of the selected studies.

### 2.4. Study Selection and Data Extraction

Search results were imported into Covidence.org for deduplication and screening. Two reviewers initially examined titles and abstracts to identify eligible studies. Articles that met the criteria then underwent full-text review by the same reviewers. Discrepancies were settled through discussion with a third reviewer to reach consensus.

Six reviewers independently collected relevant data using a standardized Excel template. These data included study details such as author, publication year, country, study design, sample size, HA product specifics, and follow-up time. It also covered patient demographics like age, sex, Fitzpatrick skin type, and baseline midface volume loss. Outcomes were categorized into efficacy measures—such as Global Aesthetic Improvement Scale (GAIS), Visual Analog Scale (VAS), and 3D volumetric analysis—and safety outcomes, including bruising, swelling, nodules, and vascular complications. This thorough approach ensured consistent and comprehensive data collection for analysis.

### 2.5. Risk of Bias and Certainty of Evidence

The risk of bias was systematically evaluated using the Cochrane ROBINS-I tool for non-randomized studies [14] and the RoB 2.0 tool for randomized controlled trials (RCTs) [15]. These tools assessed critical domains including selection bias, confounding factors, classification of interventions, deviations from intended interventions, missing data, measurement of outcomes, and selective reporting of results. Two independent reviewers conducted the assessments, with any discrepancies resolved through discussion or consultation with a third reviewer.

### 2.6. Statistical Analysis

All statistical analyses were performed using Review Manager (RevMan) software version 5.4. For continuous outcomes such as volume retention or aesthetic improvement scores, we calculated mean differences (MD) with 95% confidence intervals (CI). For dichotomous outcomes including adverse event rates and patient satisfaction measures, we computed risk ratios (RR) with 95% CI. Heterogeneity was assessed using the I^2^ statistic, with values interpreted as follows: 0–40% indicating low heterogeneity, 30–60% moderate heterogeneity, 50–90% substantial heterogeneity, and 75–100% considerable heterogeneity. A random-effects model was employed when significant heterogeneity was present (I^2^ > 50%); otherwise, a fixed-effects model was used.

## 3. Results

### 3.1. Search Results and Study Selection

**Figure 1:**
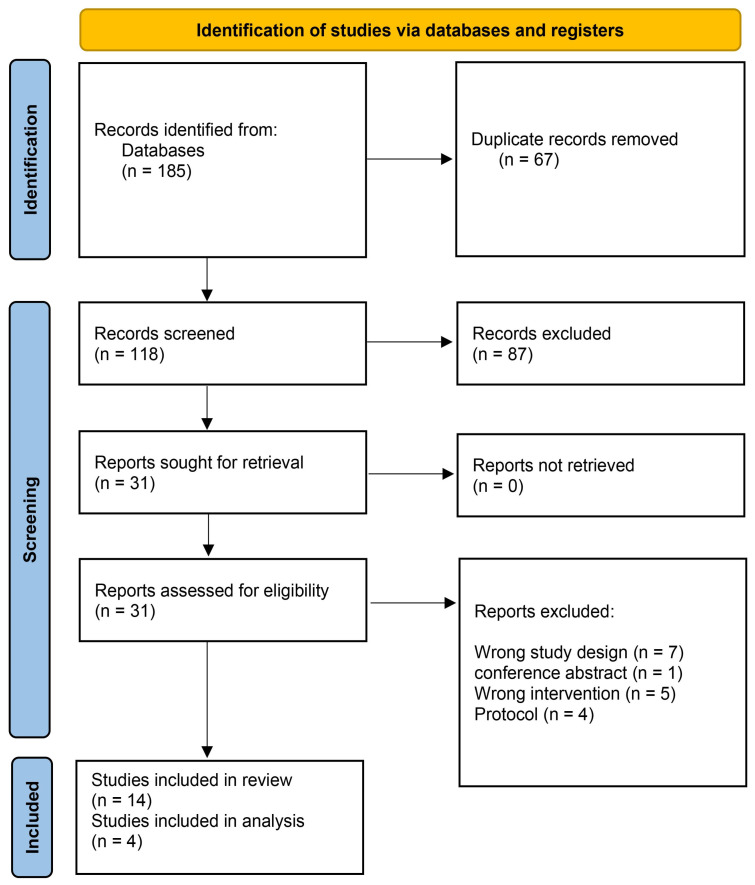
The Prisma chart used for Data extraction.

A total of 185 studies were collected from five databases into Covidence. After removing 67 duplicates, 118 records remained for screening. Of these, 87 were excluded as irrelevant during the title and abstract review. This process left 31 studies for full-text examination, and 14 of these were deemed eligible for data extraction (Figure 1).

### 3.2. Study Characteristics

The included studies comprised 14 RCTs [16,17,18,19,20,21,22,23,24,25,26,27,28,29] with a total of 1091 patients. Detailed characteristics of the included studies and study participants are presented in Table 1.

### 3.3. Quality Assessment

**Figure 2:**
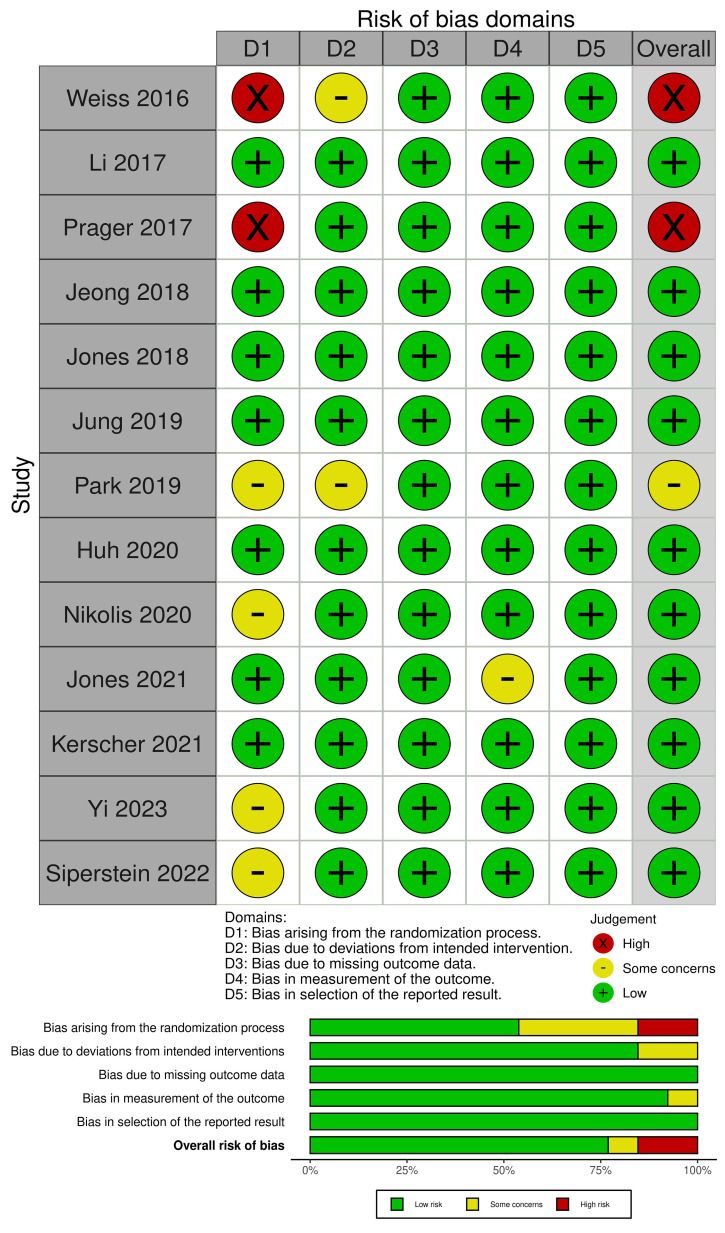
The risk of Bias assessment [16,17,18,19,20,21,22,23,24,25,26,27,28].

ROB2 assessment showed that Weiss et al. and Prager et al. had an overall high risk of bias, while Park et al. had some concerns [24,25,27]. However, the rest of the studies had an overall low risk of bias (Figure 2).

### 3.4. Meta-Analysis

#### 3.4.1. Global Aesthetic Improvement Scale (GAIS) Responder Rate

**Figure 3:**
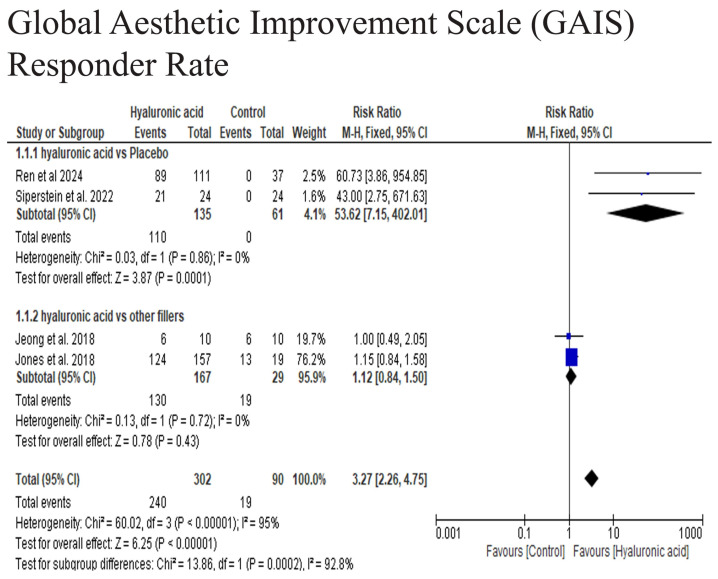
The Global Aesthetic Improvement Scale GAIS Responder Rate [17,19,26,29].

The pooled analysis of three studies demonstrated a highly significant difference in responder rates between the hyaluronic acid (HA) and control groups (RR = 3.27, 95% CI: 2.26–4.75, *p* = 0.79; I^2^ = 95%), while performing subgroup analysis the HA was comparable to other fillers (RR = 1.12, 95% CI: 0.84–1.50, *p* = 0.43), while the HA was significantly superior to placebo group (RR = 53.62, 95% CI: 7.15–402.01, *p* = 0.0001). These findings suggest that HA did not significantly enhance the proportion of patients compared to other fillers in achieving clinically meaningful aesthetic improvement, while HA is highly superior to placebo in achieving high improvement in midface augmentation (Figure 3).

#### 3.4.2. Mean Change in Global Aesthetic Improvement Scale (GAIS) over Time

**Figure 4:**
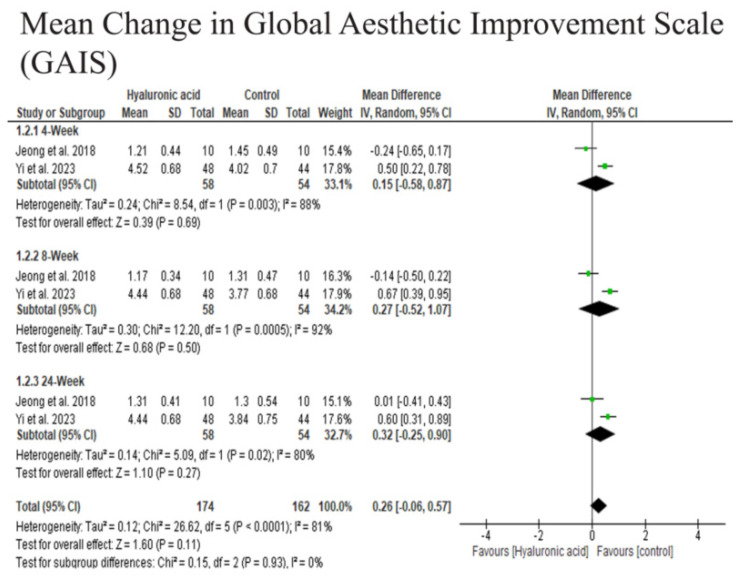
The Mean Change in Global Aesthetic Improvement Scale GAIS [17,28].

At 4 weeks, there was no significant difference in GAIS scores between the HA and other fillers (Mean Difference [MD] = −0.15, 95% CI: −0.58 to 0.87, *p* = 0.69), though substantial heterogeneity was observed (*I^2^ = 88%*). Similarly, at 8 weeks, no significant difference was found (MD = −0.27, 95% CI: −0.52 to 1.07, *p* = 0.50), with very high heterogeneity (*I^2^ = 92%*). By 24 weeks, the difference remained non-significant (MD = −0.32, 95% CI: −0.25 to 0.90, *p* = 0.27), with moderate heterogeneity (*I^2^ = 80%*). The overall pooled estimate across all time points showed no significant improvement in GAIS scores with HA (MD = 0.26, 95% CI: −0.06 to 0.57, *p* = 0.11), despite high heterogeneity (*I^2^ = 81%*). These findings suggest that HA did not provide a statistically or clinically meaningful advantage in aesthetic improvement over time compared to the control (Figure 4). The results show that the HA filler is compared to other fillers.

#### 3.4.3. Moderate to Severe Adverse Events

**Figure 5:**
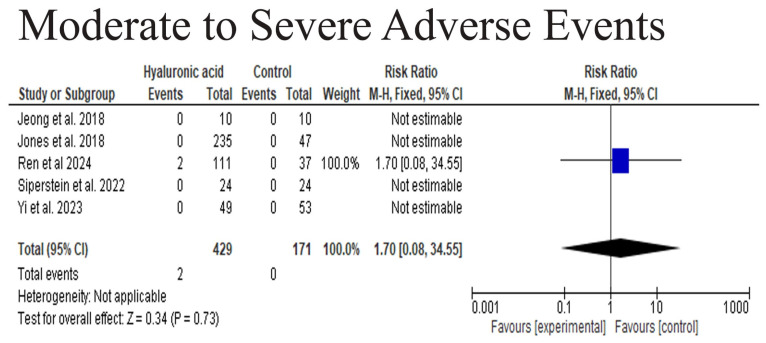
The Moderate to Severe Adverse Events [17,19,26,28,29].

The safety analysis revealed no significant difference in moderate to severe adverse events between the HA and control groups (RR = 1.70, 95% CI: 0.08–34.55, *p* = 0.73). Only two adverse events were reported in the HA group (Ren et al., 2024) [26], while none occurred in the control group. Due to the limited number of events, heterogeneity could not be assessed. These results indicate that HA was well-tolerated, with no increased risk of significant adverse effects compared to the control (Figure 5).

## 4. Discussion

This meta-analysis reports comprehensive evidence on the effectiveness and safety of hyaluronic acid (HA) fillers for midface augmentation. The combined GAIS responder rate strongly favors HA over placebo (RR = 53.62, 95% CI 7.15–402.01, *p* < 0.0001), with no heterogeneity (I^2^ = 0%), indicating a significant clinical benefit compared to no treatment. However, when HA was compared to other fillers like calcium hydroxylapatite or poly-L-lactic acid (RR = 1.12, 95% CI 0.84–1.50, *p* = 0.43, I^2^ = 0%), no notable difference was observed, suggesting similar efficacy across filler types. The overall pooled estimate (RR = 3.27, 95% CI 2.26–4.75) was significant but mainly driven by placebo-controlled trials, as indicated by high heterogeneity (I^2^ = 95%). While HA is clearly effective, its relative advantage depends on the context, as it shows superiority over a placebo but yields comparable results with other active fillers. These findings are consistent with pivotal multicenter RCTs of HA fillers, which reported significant volumetric gains and durable improvements on GAIS and related patient-reported outcomes up to 1–2 years post-injection [30,31].

A multicenter RCT demonstrated that CaHA outperforms HA in improving nasolabial folds at 8 months, and newer prospective studies suggest CaHA(+) might offer longer-lasting results than VYC-20L for midface volumization. Similarly, a multicenter randomized study by Ting et al. (2024) [32] comparing PDLLA with HA showed comparable volume correction. These results imply that although HA fillers are highly effective, they are generally not superior to other well-established volumizing agents. HA fillers are valued for their safety, reversibility, and patient satisfaction. CaHA tends to provide longer lasting volumization, especially in the nasolabial fold area, but may have a higher risk of nodularity or delayed inflammatory reactions. PLLA stimulates collagen growth and gradually restores volume, though it often requires multiple sessions and has a delayed effect. Multiple RCTs, including Ting et al. (2024) [32], confirm that HA fillers achieve similar volume correction to these alternatives, especially in the midface. This evidence suggests that while HA may not always surpass other agents in durability, it remains a preferred choice for many clinicians and patients due to its immediate results, natural look, and reversibility.

Split-face RCTs [33,34] demonstrated that certain HA products produced greater 3D volumetric change or GAIS improvement compared with others.

Objective 3-D volumetric studies and stereophotogrammetric analyses often show measurable and sometimes lasting increases in cheek volume after HA injections, even when subjective GAIS differences are minimal or not statistically significant. Therefore, studies that demonstrate volumetric benefits can align with a neutral GAIS meta-estimate, as GAIS is an ordinal, subjective measure that may be less sensitive to subtle volumetric changes detected by 3-D imaging [24,26]. Future research would benefit from more sensitive measures, such as objective volumetric imaging or 3D stereophotography.

A safety analysis revealed only two moderate-to-severe adverse events across five RCTs, resulting in a non-significant pooled risk ratio (RR = 1.70, 95% CI 0.08–34.55, *p* = 0.73). This suggests that serious adverse events are rare in controlled settings, aligning with long-term post-marketing studies that report low rates of delayed or severe complications [35]. However, systematic reviews emphasize that vascular occlusion, vision loss, and delayed inflammatory nodules, although infrequent, remain significant risks [36]. Given the limitations of RCTs in capturing rare outcomes, large-scale safety registries and extended follow-up remain essential. These severe outcomes are infrequent and not sufficiently represented in the trials included in our meta-analysis, which explains the broad confidence intervals around our adverse event estimates and restricts definitive safety conclusions. This highlights the importance of ongoing vigilance, standardized adverse event reporting, and extended follow-up in future study trials [37]. However, the wide confidence interval for our adverse event estimate (0.08–34.55) indicates imprecision due to the limited data. This highlights the need for high-powered safety studies with standardized reporting of adverse events.

Clinically, the absence of statistically significant differences should not automatically be interpreted as a lack of efficacy. Many patients seek subtle, natural improvements, and HA fillers offer a customizable, reversible, and safe option. Nonetheless, clinicians should balance patient expectations, product choice, and injection technique, recognizing that aesthetic improvements over controls may be modest in the short term. Surveys indicate that 78% of patients prefer a natural-looking, refreshed result, highlighting the importance of even subtle adjustments [38]. While our meta-analysis found no significant increase in moderate-to-severe adverse events with HA fillers, it is essential to note that rare but serious complications such as vascular occlusion, skin necrosis, and vision loss can occur. These events, although rare, underscore the importance of thorough anatomical knowledge, meticulous injection technique, and prompt recognition and management of complications. The limited reporting of severe adverse events in RCTs likely reflects their rarity rather than absence and highlights the value of large-scale safety registries and long-term post-marketing surveillance. Future studies should include extended follow-up and standardized adverse event reporting to better characterize the long-term safety profile of HA fillers.

The strengths of this meta-analysis include the comprehensive search strategy, strict inclusion criteria, and low heterogeneity in the primary efficacy results. Our updated manuscript includes a broader and more transparent search strategy, a more precise explanation of the statistical methods, and a discussion of objective outcome measures where available. We have also significantly expanded the discussion to include more in-depth comparisons with other filler types, detailed safety considerations, and practical clinical implications.

Importantly, our meta-analysis now includes several recent RCTs published up to 2024 that have not been synthesized in earlier reviews, making this the most up-to-date and comprehensive analysis on HA fillers for midface augmentation. Limitations involve the small number of eligible studies, varying follow-up times, and reliance on subjective outcome measures. Publication bias assessment was limited due to the small study pool. Additionally, the inclusion of different HA formulations and injection techniques may have added to clinical heterogeneity. A key limitation of the current literature, and thus our meta-analysis, is the reliance on subjective measures like the GAIS, which are prone to observer bias and may miss subtle volumetric changes.

The findings of this meta-analysis have important clinical implications. Patient selection, injection technique, and filler properties are crucial for achieving optimal results. HA fillers are ideal for patients seeking subtle, natural enhancement with minimal downtime, while alternatives like CaHA or PLLA may offer longer-lasting results. Clinicians should tailor treatment to the patient’s goals and anatomy and set realistic expectations about outcomes and durability.

Future trials should focus on objective volumetric endpoints, more extended follow-up periods, and standardized photographic or imaging protocols. Comparative research between HA fillers and other volumizing agents, like calcium hydroxylapatite or poly-L-lactic acid, would also be beneficial [39]. The 10-year post-marketing safety study highlights the importance of long-term follow-up and adverse event monitoring. Safety registries that track rare but serious complications continue to be essential [35].

## 5. Conclusions

HA fillers greatly enhance midface aesthetics compared to a placebo, but they do not outperform other fillers, showing similar effectiveness across different products. The safety results were positive, with mostly minor side effects and rare severe complications. Overall, HA fillers are effective, safe, and reversible, although future research should focus on objective volumetric results, longer follow-ups, and head-to-head trials with other fillers.

## Figures and Tables

**Table 1:** Detailed characteristics of the included studies and study participants.

Study ID	Place of Study	Study Design	Trial Registration Number	Sample Size	Age (Years)	Gender (M/F)	Group 1 (Intervention)	Volume of Injection	Site of Injection	Outcomes Assessed	Conclusion	Study Follow Up
Weiss 2016 [27]	USA	RCT	NA	200	52.9 ± 7.6 mean	17/183	LGP-HAL	3.8 mL	Midface (supraperiosteal to subcutaneous layer)	GAIS score, FACE-Q Score, MMVS	The treatment was safe and effective for correcting midface volume loss using LGP-HA-L	2, 14 weeks, 12 months
Li 2017 [22]	China	RCT	NA	116	20–67 median	6/140	Volume	1 mL	Deep subcutaneous/sub-periosteal (medial malar region)	Midface volume, GAIS score, patient satisfaction	Juvéderm Voluma is effective and well tolerated for midface (malar) augmentation in Chinese subjects. Significant volume increase (1.83 mL vs. 0.11 mL at 6 months, *p* < 0.001); high responder rates (98.2% by investigators, 93.8% by subjects); mild, localize	1, 6 months
Prager 2017 [25]	Germany	RCT	NA	45	38–66 median	2 Male 43 Female	CPM-26 (25 mg/mL HA)	2 mL	Subdermal/periosteal	GAIS score	Both CPM-26 and VYC-20 were effective and well-received, with a consistent trend favoring CPM-26 based on patient-reported outcomes.	3, 12, 18 months
Jeong 2018 [17]	Korea	RCT	NA	10	32–60 median	0/10	Mono-HA filler	0.6 mL	Deep dermis	GAIS score, Moire topography, adverse events	Both novel biphasic and monophasic hyaluronic acid fillers showed similar effectiveness and safety for malar enhancement over 24 weeks, with only mild and transient injection site reactions; MRI proved useful for assessing filler distribution and volume changes	2, 4, 8, 12, 24 weeks
Jones 2018 [19]	USA	RCT	NA	20	58.4 ± 10.4 mean	0/20	SP-HAL	1 mL	Intradermal microdroplet	Cheek volume, patient satisfaction	One session of intradermal microdroplet injections of SP-HAL to the mid to lower cheek failed to show superiority over normal saline in improving skin wrinkling and elastosis; procedure was safe, with only mild, transient side effects	7, 14, 28, 90, 180 days
Jung 2020 [20]	Korea	RCT	NCT02721368	83	35–65 median	19/64	Neuvamis	1 mL (max per side)	Not mentioned	GAIS score	Volume Lidocaine was non-inferior to VYC-20L for temporary midface volume restoration at 24 weeks; both fillers were effective, safe, and well tolerated with minimal safety concerns	0, 4, 12, 24 weeks
Park 2019 [24]	South Korea	RCT	NA	9	30–80 median	NA	Belotero HA filler	0.5 mL	Midface region	GAIS score, MFV scale	Monophasic fillers (B, J) demonstrated longer-lasting midface volumization than biphasic fillers (R, Y). Filler B showed excellent performance and injectability, especially in high-pressure areas, supported by objective 3D imaging data.	2, 4, 12, 24 weeks
Huh 2020 [16]	Korea	RCT	NCT02119780	68	20–65 median	NA	YVOC (22 mg/mL HA)	4.0 mL (each side)	Anteromedial malar	GAIS score, MFV score	The HA fillers injected for the anteromedial malar augmentation maintained the volume well for up to 52 weeks. Additionally, both YVOC and RESS show similar effectiveness and safety profiles	2, 14, 28, 52 weeks
Nikolis 2020 [23]	Canada	RCT	NA	30	30–75 median	0/30	Restylane Lyft (HAL)	2× per session	Not mentioned	MFVDS score, GAIS score, patient satisfaction	The use of a treatment algorithm may improve outcomes for patients seeking injectable treatments for midfacial volume loss and contour deficiencies	2, 8, 16 weeks
Jones 2021 [18]	USA (New York)	RCT	NA	210	24–80 median	24/176	HARC	6.0 mL (each side)	Subperiosteal/subcutaneous (midface)	GAIS score, FACE-Q Score	HARC&nbsp;was well tolerated and non-inferior to Control for correction of midface fullness at 12 weeks after last injection. Aesthetic improvement and subject satisfaction were high and lasted through week 48	12, 24, 36 weeks
Kerscher 2017 [21]	Germany	RCT	NA	45	18–63 median	1 Male 44 Female	CPM-26	2 mL	Not mentioned	Photographic assessment, GAIS scores	CPM-26 was non-inferior to VYC-20 based on MAS ratings at M3 and demonstrated a favorable safety and effectiveness profile for midfacial volume enhancement with results lasting up to M18.	1, 8, 12, 18 months
Yi 2023 [28]	Korea	RCT	NA	92	46.5 ± 8.72 mean	4 Male 98 Female	Giselleligne (multilayered HA)	NA	Midface Region	MFVDS score, GAIS score, operator satisfaction	This study showed that HAVOL is effective and well tolerated for midface treatment in a Chinese population	1, 4, 8, 12, 24 weeks
Siperstein 2022 [29]	US	RCT	NA	15	42.1 mean	4 Male 11 Female	VYC-17.5 L (a hyaluronic acid filler)	1 mL per side	Midface region	GAIS score, MMVS score, subject satisfaction, effectiveness and safety of HAVOL in the treatment of midface volume deficit and/or midface contour deficiency	Giselleligne is a safer, more user-friendly, and more effective alternative to existing products for improving the midfacial volume.	1, 3, 6, 9, and 12 months post-injection
Ren 2024 [26]	China	RCT	NCT03289052	148	41.3 ± 10.1 mean	12/136	HA_VOL (Restylane^®^ Volyme) group	8 mL (Total)	Midface region	GAIS score, MMVS score, subject satisfaction, effectiveness and safety of HAVOL in the treatment of midface volume deficit and/or midface contour deficiency	Giselleligne is a safer, more user-friendly, and more effective alternative to existing products for improving the midfacial volume.	1, 3, 6, 9, and 12 months post-injection

## Data Availability

Data supporting the findings of this study are available within the article.
